# Benefits of combining Metacognitive Training (MCT) with Cognitive Remediation (CR) in the recovery of patients with psychotic spectrum disorders: Preliminary results

**DOI:** 10.1192/j.eurpsy.2025.416

**Published:** 2025-08-26

**Authors:** M. Korniyenko, J. Navarro, F. González-Higueras, J. Cid, E. Frigola-Capell, I. Birules, C. Vidal, G. Garrido, J. Crosas, A. Aznar, C. Palma-Sevillano, A. Sastre-Buades, J. Sevilla-Llewellyn-Jones, O. Vallina, A. Barajas

**Affiliations:** 1 Autonomous University of Barcelona, Barcelona; 2 Hospital Universitario de Jaén, Jaén; 3 Institut d’Investigació Biomèdica de Girona Dr. Josep Trueta (IdiBGI), Institut d’Assitència Sanitària, Girona; 4 Parc Sanitari Sant Joan de Déu; 5 Fundació els Tres Turons; 6 Consorci Sanitari de Terrassa; 7 Corporació Sanitària Parc Taulí; 8 Asociación Centro de Higiene Mental Les Corts; 9 Universitat Ramon Llull, Barcelona; 10 Consorci Sanitari del Maresme, Mataró; 11 Hospital Universitario Son Llatzer, Palma; 12 Hospital Clínico San Carlos, Madrid; 13 Servicio Cántabro de Salud, Torrelavega; 14 Serra Húnter Programme, Government of Catalonia, Barcelona, Spain

## Abstract

**Introduction:**

Psychotic disorders are a major cause of global disability. While antipsychotic treatments are effective, their impact is limited. Metacognitive Training (MCT) reduces positive and negative symptoms, but neurocognitive deficits hinder therapy. Cognitive Rehabilitation (CR) may help improve these skills. Combining both therapies could offer better results, but studies are lacking to confirm whether there is any real improvement.

**Objectives:**

Compare the efficacy of combined CR+MCT therapy vs. MCT alone in clinical and functional recovery in nonaffective psychotic disorders.

**Methods:**

This ongoing randomized trial includes 85 patients (56.5% female, mean age 40.40±10.17), with 38 receiving CR+MCT and 47 receiving MCT only. Sociodemographic and clinical data (WHO-DAS-II, PANSS, GAF, and criteria for clinical remission and functional recovery) were collected pre-and post-treatment. Generalized linear models were used, with post-treatment scores as the dependent variable, baseline scores, and RC+MCT group as covariates.

**Results:**

No significant differences were found between groups. However, CR+MCT showed a greater reduction in positive symptoms (*M*_post-pre_ = -3) vs. MCT (*M*_post-pre_ = -2.2) with no changes in negative symptoms. CR+MCT presented a higher percentage of clinical remission (12,1%) vs MCT (0%) post-treatment. Both groups improve in functional recovery, with greater results in MCT alone (10,9%_CR+MCT_ vs 22,8%_MCT_). CR+MCT also had greater reductions in functional disability (*M*_post-pre_ = -3,4) vs. MCT alone (*M*_post-pre_ = -2,2).

**Conclusions:**

The group that has received the combined RC+MCT therapy has shown better results in clinical remission and functional recovery, the last in terms of disability, than the MCT-only group. The small sample size limits statistical significance.

**Disclosure of Interest:**

None Declared

